# Assessing the implementation of a multi-component hypertension program in a Guatemalan under-resourced dynamic context: an application of the RE-AIM/PRISM extension for sustainability and health equity

**DOI:** 10.1186/s43058-024-00560-5

**Published:** 2024-03-15

**Authors:** Alejandra Paniagua-Avila, Rachel C. Shelton, Juan Carlos Figueroa, Ana Lissette Guzman, Laura Gutierrez, Diego Rolando Hernandez-Galdamez, Juan Manuel Ramirez, Javier Rodriguez, Vilma Irazola, Manuel Ramirez-Zea, Meredith P. Fort

**Affiliations:** 1 Epidemiology Department, Mailman School of Public Health, Columbia University, New York, NY USA; 2 INCAP Research Center for the Prevention of Chronic Diseases (CIIPEC), Institute of Nutrition of Central America and Panama (INCAP), Guatemala City, Guatemala; 3 Department of Sociomedical Sciences, Mailman School of Public Health, Columbia University, New York, NY USA; 4 Department of Epidemiology, Dornsife School of Public Health, Urban Health Collaborative, Drexel University, Philadelphia, PA USA; 5 Institute for Clinical Effectiveness and Health Policy (IECS), Buenos Aires, Argentina; 6 Colorado School of Public Health, Anschutz Medical Campus, Aurora, CO USA

**Keywords:** Implementation science, Hypertension, Latin America, Low- and middle-income countries, Mixed methods, RE-AIM/PRISM, Health equity, Sustainability, Dynamic context, Adaptations, Hybrid trial

## Abstract

**Background:**

The COVID-19 pandemic necessitated rapid changes in healthcare delivery in Guatemala’s public primary care settings. A new hypertension program, implemented as part of a type 2 hybrid trial since 2019, exemplifies an implementation effort amidst a changing context in an under-resourced setting. We assessed the implementation of an evidence-based intervention (EBI; protocol-based hypertension treatment) and one of its main implementation strategies (team-based collaborative care), raising implications for health equity and sustainability. We present innovative application of systems thinking visuals.

**Methods:**

Conducting a convergent mixed methods analysis, we assessed implementation in response to contextual changes across five Ministry of Health (MoH) districts at the pandemic’s onset. Utilizing quantitative programmatic data and qualitative interviews with stakeholders (*n*=18; health providers, administrators, study staff), we evaluated dimensions of “Reach, Effectiveness, Adoption, Implementation and Maintenance,” RE-AIM (Reach, Implementation delivery + adaptations), and “Practical Robust Implementation and Sustainability Model,” PRISM (Organizational perspective on the EBI, Fit, Implementation and sustainability infrastructure) frameworks. We assessed representativeness by comparing participants to census data. To assess implementation delivery, we built behavior-over-time (BOT) graphs with quantitative programmatic data (July 2019–July 2021). To assess adaptations and contextual changes, we performed matrix-based thematic qualitative analysis. We converged quantitative implementation delivery data + qualitative adaptations data in joint displays. Finally, we analyzed qualitative and quantitative results across RE-AIM/PRISM and health districts to identify equity and sustainability considerations.

**Results:**

Contextual factors that facilitated program delivery included the perception that the EBI was beneficial, program champions, and staff communication. Key barriers to implementation delivery included competition with other primary care activities and limited implementation infrastructure (e.g., equipment, medications). Contextual changes related to COVID-19 hindered implementation delivery, threatened sustainability, and may have exacerbated inequities. However, adaptations that were planned enhanced implementation delivery and may have supported improved equity and sustainability.

**Conclusions:**

Recognition of an EBI’s benefits and program champions are important for supporting initial uptake. The ability to plan adaptations amid rapid contextual changes has potential advantages for sustainability and equitable delivery. Systems thinking tools and mixed methods approaches may shed light on the relations between context, adaptations, and equitable and sustainable implementation.

**Trial registration:**

NCT03504124

**Supplementary Information:**

The online version contains supplementary material available at 10.1186/s43058-024-00560-5.

Contributions to the literature

- The COVID-19 pandemic offered an opportunity to learn how contextual changes influence the adaptation, equitable and sustainable implementation of evidence-based interventions (EBIs), and implementation strategies.
- Our implementation assessment describes how Ministry of Health (MoH) frontline primary care teams in rural, low-resource settings in Guatemala adapted to contextual changes to implement a new hypertension program, before and during the initial phase of the COVID-19 pandemic.
- This article shows how mixed-methods joint displays may combine behavior-over-time (BOT) graphs, a systems thinking tool, to display the dynamic nature of implementation over time, with qualitative quotes showcasing adaptations to the intervention.

## Background

Understanding the interplay between evidence-based interventions (EBIs), implementation strategies and multi-level context is essential to shed light on the nature of successful implementation efforts [1]. Several Dissemination and Implementation Science frameworks recognize the influence of dynamic, multi-level contexts on the implementation of EBIs and their implementation strategies [2, 3]. Such frameworks highlight that EBIs are not discrete packable units that can be implemented in the same way in every setting [4]. Such frameworks also question the idea that interventions can be fully optimized during the pre-implementation phase and then implemented with complete fidelity [4]. Instead, EBIs and implementation strategies are seen as adaptable in response to changes within context and over time [2, 5]. Learning from real-world case experiences allows for a better understanding of the interconnections between context, EBIs and implementation strategies, and may inform future implementation experiences [6]. Following calls for addressing evidence gaps in implementation science, it is particularly important to study implementation in the context of under-resourced settings in low- and middle-income countries (LMICs).

In recent years, there has been an increasing interest in integrating sustainability and health equity perspectives into implementation assessments of EBIs [5, 7–9]. Following principles of the Dynamic Sustainability Framework, “Reach, Effectiveness, Adoption, Implementation and Maintenance” (RE-AIM) framework extension was developed to integrate sustainability and health equity into the assessment of implementation outcomes within dynamic contexts [10]. Sustainability is the extent to which an EBI continues to be delivered and continuously delivers its intended benefits over an extended period [10, 11]. Assessing sustainability involves documenting the functions of an EBI and implementation strategies across their life cycle [10]. Health equity is seen as the fair and just opportunity to be as healthy as possible [10]; equitable implementation requires documenting and addressing inequities as they emerge [10]. Importantly, the RE-AIM extension for sustainability recommends the repetitive evaluation of sustainability and health equity from the early implementation phases [10]. This framework may be combined with its contextual expansion, the “Practical Robust Implementation and Sustainability Model” (PRISM) framework, to capture the multi-level contextual factors influencing sustainability and health equity [3, 12, 13].

In 2020, the COVID-19 pandemic emerged, requiring drastic and rapid changes to healthcare settings and communities at large, overburdening already strained systems and healthcare workers, in high-income as well as LMICs [14]. Under-resourced settings within LMICs have been disproportionally affected due to fragile primary care systems and limited availability of primary care workers. In these settings, the COVID-19 pandemic threatened the sustainability of EBIs for chronic health conditions and highlighted pre-existing health and social inequities [14–16]. Worldwide, the COVID-19 pandemic provided a striking example of a crisis that provoked significant changes to dynamic contexts. To implementation scientists, the pandemic offered an opportunity to better understand how contextual changes influenced the implementation and adaptation of EBIs and implementation strategies. Following calls for developing evidence and learning from implementation experiences in LMICs [6, 17, 18], we conducted an assessment of the implementation of a multi-component hypertension program in rural Guatemala.

Since 2018, our team has been studying the implementation and effectiveness of a multi-component hypertension program (thereafter referred to as “program”) delivered within the public primary care system in rural Guatemala [19]. In 2018, we conducted qualitative formative research to assess needs and adapt the program to the local context [20, 21]. In 2019, we launched the program implementation and assessment through a hybrid type 2 cluster randomized control trial (cRCT). This implementation initiative has been led by the Institute of Nutrition of Central America and Panama (INCAP) in partnership with Guatemala’s Ministry of Health and Social Welfare (MoH) [22]. The program is a multilevel and multicomponent intervention that consists of a core EBI and five evidence-based implementation strategies [19–21]. The core EBI is a protocol-based stepped care hypertension treatment. The implementation strategies are team-based collaborative care, health provider training, health coaching sessions, home blood pressure monitoring, and blood pressure audit and feedback. The program has been implemented within public primary care facilities in rural Guatemala: health posts (first level of primary care) and health centers (second level of primary care) (See “ Study setting” below). A detailed description of the program and the study protocol is published elsewhere [19].

In March 2020, 9 months after initiating program implementation, COVID-19 cases were on the rise in Guatemala and a national-level response with lockdowns and significant disruptions to rural communities and healthcare services occurred [23]. As in other LMICs, Guatemala’s public primary care services were disrupted and required rapid and drastic changes, providing a noteworthy example of a rapidly evolving context. During this period, we conducted our first planned implementation assessment of the program, utilizing the contextually expanded RE-AIM/PRISM framework [24].

The aim of this assessment was to describe the implementation delivery of the hypertension program and its adaptations in response to contextual changes within the public primary care system in rural Guatemala during the COVID-19 pandemic. We present implications for the sustainable and equitable implementation of the program.

## Methods

### Study setting

This study took place within the public primary care system which serves 70% of the population and is governed by the Guatemalan MoH. This system is organized into two levels of care: the first level of primary care and the second level of primary care. The first level is a network of health posts, run by auxiliary nurses that provide preventative and primary care services at the community level. The second level takes place within the health district’s municipal health center, which is run by auxiliary nurses, professional nurses, and primary care doctors. Providers in the second level of primary care supervise those in the first level and manage more complex cases that are referred from the first level of primary care. The parent cRCT took place in 36 municipal health districts (clusters), half of which received the intervention program. Auxiliary nurses were primarily responsible for delivering the program with supervision from professional nurses and, in some districts, support from general doctors. At the time of this assessment, eight of the 18 intervention districts had initiated program implementation. To capture implementation and contextual changes before and after COVID-19, we conducted this assessment in the eight districts with the longest experience implementing the program. Results from the final implementation assessment including the 18 intervention districts will be published elsewhere.

### Implementation science frameworks

We utilized the RE-AIM extension for sustainability and the PRISM framework to guide this assessment (See Table 1) [24, 25]. RE-AIM includes five implementation outcomes—reach, effectiveness, adoption, implementation and maintenance—at the participant and implementer levels, critical to producing widespread implementation and broad population health impact [25–27]. In this assessment, we focused on RE-AIM’s reach and implementation dimension, respectively operationalized as representativeness of hypertensive patients and implementation delivery + adaptations [28]. The RE-AIM extension on sustainability was pertinent given that the COVID-19 emergency led to rapid contextual changes, providing an opportunity to document implications for health equity and sustainability in relation to implementation. PRISM, a contextual expansion of RE-AIM, was also used here to identify the multi-level factors that influenced implementation delivery + adaptations, including fit between the intervention and context, and implementation and sustainability infrastructure [24, 29].

**Table 1:** Study summary box. Overview of mixed methods implementation assessment: implementation frameworks, selected dimensions, qualitative questions / quantitative indicators, method, data sources and participants / settings

**Framework: selected dimension**	**Qualitative questions / quantitative indicators**	**Method: data sources**	**Participants / settings**
**RE-AIM:** **Reach**	Representativeness: Are characteristics of study health facilities / communities representative of the larger population?	Quant: Programmatic data; census data	Municipal health centers and health posts
**RE-AIM: Implementation**	Implementation delivery: Are key program indicators (hypertensive medications, team meetings) consistently delivered over time?	Quant: programmatic data	Municipal health centers and health posts
	Why and how was the program adapted?	Qual: In-depth interviews	Study staff, MoH providers and administrators
**PRISM: Organizational perspective on the program**	What are the perspectives of health district’s providers and administrators on the program?	Qual: In-depth interviews	Study staff, MoH providers and administrators
**PRISM: Fit between program and health district**	How did health district-level factors facilitate or hinder the program implementation?	Qual: In-depth interviews	Study staff, MoH providers and administrators
**PRISM: Implementation and sustainability infrastructure**	What needs to be available at health facilities for the program to be implemented and sustained?	Qual: In-depth interviews	Study staff, MoH providers and administrators
**Health equity considerations**	What are the potential sources of unequal implementation between health facilities and community subgroups? What type of program adaptations may reduce or promote equitable implementation?	Qual: In-depth interviews Mixed methods: Meta-inferences across qual and quant results	Study staff, MoH providers and administrators All participants and settings
**Sustainability considerations**	What contextual changes may threaten program sustainability? What type of program adaptations may influence its implementation delivery and its sustainability?	Qual: In-depth interviews Mixed methods: Meta-inferences across qual and quant results	Study staff, MoH providers and administrators All participants and settings

*MoH* Ministry of Health, *N/A* not applicable

### Overview of study design

Frameworks, selected dimensions, questions, and data sources are summarized in Table 1. We used a convergent mixed methods design to assess implementation of program in municipal health centers and health posts [30]. Specifically, we assessed the EBI core component (protocol-based stepped care hypertension treatment) and one of its main implementation strategies (team-based collaborative care). Quantitative data on participant enrolment was used to assess reach. Programmatic data was used to assess implementation delivery at the healthcare facility level. Qualitative in-depth interviews with stakeholders were used to assess RE-AIM program implementation delivery + adaptations, sustainability, health equity, and PRISM contextual implementation factors. Programmatic data was collected on a monthly basis from July 2019 to June 2021, while qualitative data was collected from July 2020 to September 2020, at the initial phase of the pandemic. This study was approved by the Guatemalan National Ethics Committee, INCAP’s Institutional Review Board (IRB), Columbia University Irving Medical Campus IRB, and the University of Colorado IRB.

### Qualitative phase

#### Interview participants

We aimed to comprehensively assess implementation from different perspectives and health system levels. Therefore, we conducted 18 interviews with individuals who were closely involved in the program implementation and had diverse responsibilities at different levels of the primary care system. First, we included MoH staff (*n*=8) who were responsible for managing and providing the program. MoH staff included administrators (*n*=4) and providers (*n*=4) from the central-level office in the capital (*n*=1), the provincial level (*n*=3), and the municipal/district level (*n*=4). Second, we included INCAP research staff (*n*=10) who were responsible for training healthcare providers and providing clinical and administrative guidance to the MoH staff as they implemented the program. INCAP research staff included research assistants working at the provincial level (*n*=5) and field researchers working at the health post / community level (*n*=5). Participants were invited to participate by APA or INCAP research assistants. All invitees agreed to participate and provided verbal informed consent prior to initiating interviews.

#### Qualitative data collection

Qualitative in-depth interviews were guided by the RE-AIM extension for sustainability and PRISM frameworks (See Table 1; See interview guides in Supplementary Material 1). We initially asked participants to describe the program in their own words and share their perspectives about it. Then, we inquired about the implementation of the EBI / implementation strategy and contextual changes before and during COVID-19, focusing on the adaptations and reasons behind such adaptations. To assess fit, we inquired about the organizational factors that influenced program implementation. To assess implementation and sustainability infrastructure, we asked about what would need to be in place for the program to be implemented and sustained during and beyond the study period. We asked about potential sources of inequitable program implementation, and ways to overcome them. Finally, participants shared lessons learned and recommendations to sustain the program beyond the study period.

Interviews were conducted by APA and ALG in Spanish, lasted 30–60 min, and were audio recorded over phone. We collected interviews in July–September 2020, 9–12 months after the program was launched, and 3–5 months into the pandemic lockdowns, at the height of the first peak of COVID-19 cases in Guatemala.

#### Qualitative data analysis

To analyze qualitative data, two independent members of the research team (APA, ALG) performed an exploratory thematic analysis following an inductive-deductive approach on all interview templates. Deductive categories followed pre-defined themes corresponding to RE-AIM / PRISM domains from the interview guides (Table 1). Inductive themes emerged from reviewing each interview summary. To facilitate the analysis, we followed the Rapid Identification of Themes from Audio Recording (RITA) methodology and the matrix-based approach to thematic analysis [31, 32]. RITA consists of identifying pre-defined themes from audio recordings without the need to have verbatim interview transcriptions and line-by-line coding [31]. Our pre-defined themes corresponded to RE-AIM / PRISM domains (See Table 1**)**. Following RITA, two analysts (APA, ALG) independently listened to interview recordings and captured the main ideas for each question in an interview summary. Interview summaries by each analyst were reviewed by one analyst (APA) and consolidated. We created a matrix with RE-AIM / PRISM domains and allowed the matrix to evolve as the team identified new themes within such categories. The team held discussions to review and agree on a final matrix (See Supplementary material 2). Two independent analysts (APA, ALG) coded interview summaries and transferred the data into the final matrix. Discrepancies were solved through discussion until no new ideas emerged indicating saturation [33]. Finally, one member of the team transferred all the data into a consolidated matrix that included all the information for each of the health district.

Analysis was conducted by a multidisciplinary team with expertise in qualitative methods (all), medicine (APA), chronic diseases (ALG), health systems (MPF), and implementation science (MPF, APA). We met weekly to discuss results and implications for health equity and sustainability. Other co-authors were consulted at key points in the analysis to share their expertise. See the Consolidated criteria for reporting qualitative research (COREQ) checklist in the Supplementary Material 3.

### Quantitative phase

#### Quantitative data collection

We used quantitative data to measure two RE-AIM dimensions: program reach and program implementation delivery over time. As we were interested in equity, we focused on the representativeness of study participants receiving the intervention as compared to the general population in the study setting.

We measured implementation delivery of both the EBI and the implementation strategy, at the healthcare facility level and on a monthly basis (July 2019 to July 2021). To assess the implementation delivery of the EBI, we documented availability of hypertensive medications per month period. To assess implementation delivery of the implementation strategy, we documented the number of team meetings per month period. Quantitative data was collected by MoH auxiliary nurses who were also in charge of delivering the program. Data was collected on paper-based forms that were previously co-designed and piloted by the research team and MoH staff. Every 3 months, INCAP study staff reviewed and entered the paper-based data into a ReDCap electronic database, which was hosted by INCAP in a centralized research site in Guatemala City [34, 35].

#### Quantitative data analysis

To assess representativeness, we compared the sociodemographic characteristics of study participants captured at the cRCT enrollment visit, to those of the general population captured in the Guatemala’s National Census 2018 [36]. To measure availability of hypertensive medications over time, we estimated the proportion of observed vs expected percentage of available hypertensive medications per 1-month period at each health district. To measure implementation delivery of the implementation strategy, we estimated the percentage of the observed vs expected number of team meetings per 3-month period at each health district. With each indicator of implementation, we built behavior-over-time (BOT) graphs displaying the implementation delivery from July 2019 to July 2021, before and during COVID-19 [37].

### Qualitative and quantitative data convergence and meta-inferences

**Fig. 1:**
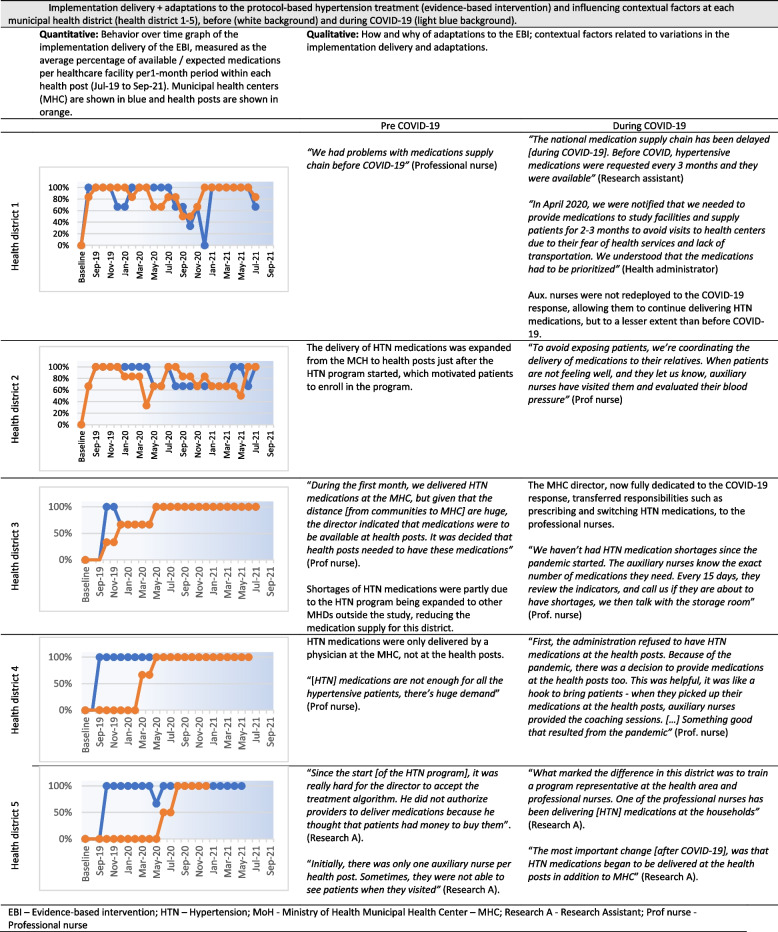
Implementation delivery + adaptations to the protocol-based hypertension treatment (evidence-based intervention) and influencing contextual factors at each municipal health district (health district 1-5), before (white background) and during COVID-19 (light blue background). *EBI* Evidence-based intervention, *HTN* Hypertension, *MoH* Ministry of Health, *MHC *Municipal Health Center, *Research A *Research Assistant, *Prof nurse *Professional nurse

**Fig. 2:**
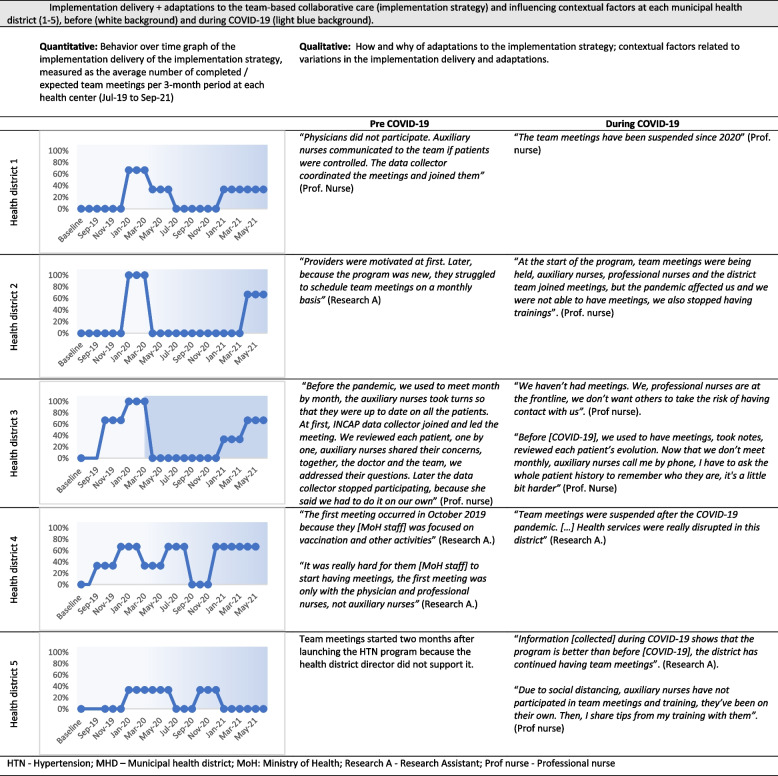
Implementation delivery + adaptations to the team-based collaborative care (implementation strategy) and influencing contextual factors at each municipal health district (1-5), before (white background) and during COVID-19 (light blue background). *HTN* Hypertension, *MHD* Municipal health district, *MoH* Ministry of Health, *Research A* Research Assistant, *Prof nurse* Professional nurse

Finally, we converged qualitative and quantitative results related to implementation delivery + adaptations by building joint displays (Figs. 1 and 2) [30, 38]. We selected illustrative quotes, themes and BOT graphs in two joint displays: one for the EBI (protocol-based hypertension treatment) and one for the implementation strategy (team-based collaborative care) [39]. We also compared and contrasted qualitative and quantitative results across RE-AIM / PRISM dimensions and health districts to reach meta-inferences related to equitable program implementation and sustainability (See Table 5).

## Results

### Overview

Table 2 presents an overview of key results organized by RE-AIM / PRISM dimensions: reach (representativeness), implementation (implementation delivery + adaptations), organizational perspectives on the hypertension program, fit between the program and the health district, implementation and sustainability infrastructure, health equity considerations, and sustainability considerations. The following paragraphs expand on major qualitative themes and key results, following Table 2.

**Table 2:** Overview of findings by selected RE-AIM and PRISM dimensions

**Framework: Selected dimension**	**Overview of results**	
**RE-AIM:** **Reach**^**a**^	Representativeness of cRCT study participants compared to the general population - Variations in sociodemographic characteristics	
**RE-AIM: Implementation**^**b**^	Stepped-care hypertension algorithm (EBI) - Implementation delivery: Variations in the availability of HTN medications before and during the COVID-19 pandemic - Adaptations: Mechanisms to supply and provide HTN medications - Adaptations: Varying roles of healthcare workers in the implementation of the HTN protocol Team-based collaborative care (Implementation strategy) - Implementation delivery: Variations in the frequency of team meetings before and during the COVID-19 pandemic - Adaptations: Frequency of team meetings and types of providers who participated	
**PRISM: Organizational perspective on the program**^**c**^	Facilitators - Perceiving the HTN program as effective or beneficial to community members engaged HCPs in the program delivery.	Barriers - Perceiving the HTN program as additional workload, an imposed activity, or complicated, hindered HCP’s engagement in the program delivery and led to lack of support from health district leadership.
**PRISM: Fit between program and health district**^**c**^	Facilitators - Previous experience providing chronic diseases services at the health district - Program champions and strong leadership at the health district- and health area-level - Healthcare team organization, collaboration and communication	Barriers - Competition between HTN program and other MoH primary care programs - Insufficient and overburdened healthcare staff - Temporary suspension of healthcare services due to COVID-19
**PRISM: Implementation and sustainability infrastructure**^**c**^	Resources - Essential equipment and supplies to deliver the program (e.g., blood pressure monitors) - Essential human resources to deliver the program together with 22 other primary care programs - Transportation for HCPs to visit patients unable to visit health facilities - Financial resources to cover chronic diseases programs, like hypertension Processes - Effective supply chain of hypertensive medications to ensure consistent access for patients - Supervisory team for chronic diseases programs, including hypertension - Training of HCPs on hypertension management - Effective health information system to capture key indicators of hypertension program	
**Health equity considerations**^**d**^	Factors related to reach and equitable implementation - Sociodemographic and community characteristics: males working in agricultural sector, poverty and unemployment, limited literacy, language barriers and ethnicity, rurality, family support, machismo, community leadership - Health district characteristics: implementation and sustainability infrastructure (e.g., program champions) - COVID-19 enhanced health inequities among subgroups (e.g., poverty, language barriers) Program adaptations related to enhanced reach and equitable implementation - Diverse forms to reach all participants (e.g., home visits to reach unemployed participants) - Diverse forms to continue reaching participants during COVID-19 (e.g., phone calls during COVID-19)	
**Sustainability considerations**^**d**^	Contextual factors related to program sustainability - Implementation and sustainability infrastructure (e.g., equipment, human resources) - Fit between program and health districts (e.g., experience w/chronic diseases program) - COVID-19 threatened program sustainability (e.g., insufficient staff, supply chain) Program adaptations related to program sustainability - Diverse forms to continue delivering program during COVID-19 (e.g., phone calls among collaborative care team in addition to in-person team meetings)	

*HCPs* healthcare providers, *HTN* hypertension

^a^See Table 2 for expanded results about program reach

^b^See Figures 1 and 2 for expanded results about program implementation delivery + adaptations

^c^See Table 3 for expanded results about contextual factors

^d^See Table 5 for expanded results about health equity and sustainability considerations

### RE-AIM: reach and implementation

#### Reach (representativeness) of patient participants

In the comparison of baseline sociodemographic characteristics of cRCT participants and the general population, we identified important details (See Table 3). The study sample had a higher percentage of people who were female, older, and married than the general population. Our study sample had a lower percentage of indigenous people compared to other ethnicities, and participants who were literate and employed, as compared to the census population for the included geographic areas.

**Table 3:** Characteristics of participants enrolled in the multi-component hypertension intervention program in rural Guatemala compared to the general population

**Variable**	**Enrolled participants**^**a**^	**General population**^**b**^
Female, %	71.0	53.2
Age, mean (SD), years	62.9 (11.4)	56.39 (12.4)
Married, %	64.6	72.2
Indigenous Maya, %	23.5	25.7
Literacy (able to read and write), %	48.6	57.9
Employed, %	27.2	45.1

^a^Enrolled participants in parent cluster randomized controlled trial (cRCT)

^b^Characteristics of general population from municipalities where study took place based on National Census 2018

#### Availability of medications to deliver the EBI (protocol-based hypertension treatment) and adaptations

As shown in Fig. 1, availability of medications to deliver the protocol-based hypertension treatment (EBI) evolved, before and during COVID-19. BOT graphs show that the availability of hypertensive medications increased shortly after launching the program within all the assessed health districts. Later, availability differed by health district. Qualitative data about the how and why of adaptations provided potential explanations for differences in BOT graphs. Taking health district 2 as an example, before COVID19, administrators worked to ensure the availability of hypertensive medications at the health center and health posts to facilitate patient access, an example of a planned adaptation needed to implement the program over time. Later, during COVID19, supply chain disruptions reduced the availability of hypertensive medications, an example of a contextual adaptation that occurred and impacted implementation. In contrast, health district 3 (HD3) had sufficient hypertensive medications prior to and during COVID19 due to frequent medication surveillance and coordination between health centers and health posts to sustain the medication supply chain, an example of a planned adaptation in the context of COVID-19.

#### Implementation delivery and adaptations to the implementation strategy (team-based collaborative care)

As shown in Fig. 2, implementation delivery of team-based collaborative care evolved over time as indicated by the completion of team meetings**.** We found quantitative and qualitative differences in implementation between pre- and during COVID19 periods. BOT graphs show that in health districts 1–3, the implementation of team meetings was greater than 60% at least once before COVID-19 but dropped to 0% during COVID19. In contrast, implementation of team meetings was consistently low at health districts 4–5 before and during COVID19. Qualitative data confirmed and expanded on the quantitative results. For instance, team meetings were suspended during COVID-19 to keep social distance at health district 3, a planned adaptation. Also, primary care services were suspended at health district 4. In contrast, at health district 5, the delivery of team meetings was routinely lower than at other health districts due to lack of program support from leadership. Qualitative data also provided details on adaptations to the implementation strategy, expanding on the quantitative information. For example, during COVID19, the BOT graph for health district 3 shows that the meetings dropped to 0%. Qualitative data, however, show that despite the suspension of team meetings, this health district continued carrying out the function of the team-based collaborative care strategy, because healthcare providers continued coordinating with each other by phone in order to provide team-based collaborative care, an example of a planned adaptation in the context of COVID19.

### PRISM: multi-level contextual factors influencing program implementation

#### Organizational perspectives on the hypertension program

We asked participants to describe the program and share their perceptions about it (See Table 4). We found that the perspectives of healthcare providers / administrators (i.e., the implementers) appeared to influence program implementation (implementation delivery + adaptations) and the intention to sustain it. Perspectives varied widely between health districts and the type of healthcare provider / administrator. In health district 5, lack of support from leadership delayed the initiation of team-based collaborative care meetings (implementation strategy) and other program elements. At health district 1, an observed facilitator was that healthcare administrators and nurses perceived it as “beneficial to community members” and a “learning opportunity.” Whereas in that same district, a barrier was that health district 1 physicians perceived the program to be an addition to their workload and they were minimally involved in program delivery. Program implementation was adapted to the context; professional nurses took on physicians’ responsibilities, such as evaluating patients, prescribing medications, and coordinating team-based collaborative care. These adaptations allowed the program to be implemented in this district, overcoming the lack of support from physicians. If healthcare providers and administrators perceived the program as beneficial and feasible, they appeared to support its institutionalization (with implications for sustainment) within the MoH beyond the study period.

**Table 4:** Representative quotes for selected PRISM dimensions

**PRISM dimension**	**Representative quotes (Role of interviewee, municipal health district)**
**Organizational perspective on the program**	“Physicians did not buy into it; they did not participate in the program and some staff perceived the program as “additional workload”. Professional nurses ended up taking on responsibilities that had been assigned to physicians.” (Data collector, MHD1) “I think that the MoH can continue [implementing the program], as it can help patients and prevent [hypertension] […]. Patients go back home and share their experiences with their relatives on how to prevent chronic diseases – diet, salt consumption, weight, exercise, many habits that should be avoided, like eating junk food. This is very important and should be sustained. The MoH should take it on as its own program” (Health district administrator, MHD3)
**Fit between program and health district**	**Competition with other MoH primary care programs** “During the initial phase [of COVID-19], services for patients with chronic diseases were suspended, all at once we had 13 COVID cases, we were afraid.” -Professional nurse, HD3 “Remember that an auxiliary nurse is responsible for 22 other MoH programs, and now, with this pandemic it’s even worse.” (Professional nurse, health area administration for MHD 1 and 2) **Previous experience providing services for chronic diseases** “This isn’t a new program for us. Since 2011 we’ve been following-up with patients with chronic diseases, monthly or annually, as part of the Inclusive Health Model (Modelo Incluyente de Salud, MIS), which is supported by the Institute for Inclusive Health. […] Implementing this program has not been very hard for our district. This new program improved our approach to managing hypertension.” (Professional nurse, MHD1 administrator) **Program champions and strong leadership** “The health area administrator really supports the program and she has made sure that hypertensive medications are available, which motivates patients to participate in the program.” (Data collector, MHD2)
**Implementation and sustainability infrastructure**	**Human resources** “Our main challenge to deliver this program is that we need more human resources.”** (**Health area nurse, MHD1 and 2) **“**The program can be implemented as planned, but there needs to be a supervisory team from the MoH in charge of monitoring the program, supervising and training [healthcare providers].**” (**Research assistant, MHD3) **Essential equipment and hypertensive medications** “This program has been possible just because INCAP provided us with blood pressure monitors, weight balances…” (Professional nurse, health area administration for MHD 4 and 5)

*MHD* Municipal health district

#### Fit between hypertension program and health districts

We asked participants to explain how the characteristics of health districts influenced program implementation (fit), which we categorized into barriers and facilitators (See Table 4). Most participants indicated that competition with other MoH primary care program activities was a major barrier to program fit. Two other barriers identified by most participants were: insufficient and overburdened healthcare staff and the temporary suspension of healthcare services early in the COVID-19 pandemic. Facilitators related to fit were: prior experience providing services for chronic diseases, the presence of program champions and strong leadership, and the extent of organization, collaboration and communication within the district team. At the time that the interviews were conducted, the number of COVID-19 infections were on the rise and providers were increasingly becoming involved in the pandemic response, which presented an additional challenge for program fit across health districts.

##### Competition with other MoH primary care programs

Across all health districts, all participants described the challenges related to delivering the program in addition to 22 other MoH primary care programs (See Table 4). Most providers described the tension between delivering care for acute problems and maternal and child health programs as compared to care for chronic conditions, such as hypertension, as historically, maternal and child health programs have been prioritized over chronic diseases. Most participants indicated that providers had to choose between delivering one program over the other one. For example, a data collector at health district 2 described that providers had to team meetings to respond to urgent problems (e.g., an undernourished child), reducing the delivery of this implementation strategy. Moreover, the COVID19 pandemic response initially led to the suspension of healthcare services for chronic diseases, and later created more responsibilities among the primary care team, reducing program fit for the district.

##### Prior experience providing services for chronic diseases

Most participants suggested that program fit was facilitated by the health district’s experience providing care for chronic diseases (e.g., diabetes mellitus 2 and hypertension) (See Table 4). MoH administrators highlighted that health districts with previously existing chronic disease programs had capacities and facilitators to implement the hypertension program. For instance, the healthcare team was already trained in chronic diseases, accustomed to, and organized in such a way that allowed them to follow up with chronically ill patients. In contrast, other healthcare teams were only organized to provide care for acute health problems (see previous theme).

##### Program champions and strong leadership

Most participants (MoH and study staff) across health districts highlighted that strong leadership and program champions enhanced program fit in the health districts (See Table 4). Program champions were MoH leaders, such as health area or health district administrators, who strongly supported the hypertension program and mobilized their teams to implement it. They also actively adapted the EBI / implementation strategy and ensured that key infrastructure elements were available, which enhanced the program’s fit to their health district (see implementation and sustainability infrastructure below). For example, the medical director of health district 3 ensured the availability of hypertensive medications at health centers and health posts, facilitating the implementation delivery of the protocol-based hypertension treatment (see Fig. 1). Program champions also facilitated the program’s expansion and sustainability potential. For example, the medical director at health district 3 promoted the program expansion to additional health facilities, adapted it to manage diabetes mellitus 2 in addition to hypertension, and communicated the importance of sustaining the program beyond the study period to healthcare providers.

#### Implementation and sustainability infrastructure

We asked interview participants to reflect on what would be needed for the program to be implemented and sustained over time. Participants identified two essential components of the implementation and sustainability infrastructure: human resources, and equipment and medications (See Tables 2 and 4).

##### Human resources

Most participants across health districts emphasized the need for additional human resources to deliver the program in addition to the other primary care programs (e.g., childhood immunizations) (See Table 4). Some participants suggested that health districts would need at least three auxiliary nurses per health post, instead of one or two. Moreover, to sustain the program beyond the study period, participants suggested creating the role of a local-level chronic disease program coordinator, who would be in charge of patient navigation, monitoring of hypertensive medication availability, and health staff coordination between health centers and health posts. Providing ongoing training, supervision, and support to auxiliary nurses was also identified as a need by most participants.

##### Essential equipment and hypertensive medications

Most participants identified the need for equipment (e.g., blood pressure monitors) and recognized that prior to the start of the program such equipment was not available at most health facilities (See Table 4). Most participants highlighted that ensuring availability of hypertensive medications had been challenging before and particularly during COVID-19. Some participants mentioned that, to sustain the program beyond the study period, health districts would need to improve the medication supply chain.

### Health equity considerations

As we converged data to identify health equity considerations, we found that the qualitative and quantitative phases across settings, participants and RE-AIM / PRISM dimensions confirmed each other. Table 5 summarizes meta-inferences and representative quotes related to health equity considerations. First, we found sociodemographic and community characteristics related to reduced reach and implementation delivery within groups. The most salient characteristics identified by participants included men working in the agricultural sector; people living in poverty or unemployed; people with limited literacy; people who lacked family support; indigenous people who did not speak Spanish, the language used to deliver the program; women who were prohibited from participating in the program by their husbands; communities located in isolated and mountainous areas; lack of community leadership and support for the program. The overrepresentation of female participants in our sample (71.0% vs 53.2% in the general population) may indicate barriers to reach men working in agriculture. Second, we found that health district characteristics corresponding to PRISM contextual factors may have influenced whether the implementation delivery occurred was equitable across districts. For example, limited fit and limited implementation and sustainability infrastructure may have led to unequal implementation delivery between health districts (e.g., see Fig. 2, health district 3 vs 5). Third, the COVID-19 pandemic seemed to enhance pre-existing health inequities between community subgroups (e.g., low vs high community leadership) and health districts (e.g., limited implementation infrastructure). Finally, certain program adaptations seemed to enhance reach and equitable implementation. For instance, conducting home visits to deliver hypertensive medications helped to reach unemployed participants who were not able to visit health facilities. Instead, following-up participants through phone calls during COVID-19 may have inadvertently increased health inequities across the socioeconomic spectrum.

**Table 5:** Quotes representing the most salient considerations for health equity and sustainability

	**Representative quotes**	**Meta inferences**
**Health equity considerations**	Equitable implementation of future hypertensive programs would require: •Designing strategies to reach and follow-up men working in agriculture •Addressing language preferences and limited literacy •Addressing structural factors: gender roles, poverty, unemployment, community engagement •Enhancing implementation and sustainability infrastructure for hypertensive programs within municipal health districts (e.g., equipment, human resources, transportation) •Enhancing the public health response to emerging diseases (e.g., COVID-19) Most participants identified sources of health inequities at the participant- and health facility-levels	“Some female participants required husband authorization to enroll in the program. Some were not authorized and did not enroll in the program” – Data collector, MHD1 “It is easier to work with patients who have a relative who is willing to help them, if they know how to read and write, and if they live closer to the health services. Patients without family support may not receive hypertensive medications” -Data collector, MHD2
**Sustainability considerations**	Sustainable implementation of future hypertensive programs would require: • Enhancing the fit of health districts by including programs for chronic diseases within primary care settings and ensuring understanding of intervention benefits prior to implementation • Training leadership to design program adaptations in response to contextual changes (e.g., COVID-19) • Strengthening communication and collaboration among primary care teams • Ensuring essential implementation and sustainability infrastructure within health districts • Ensuring essential infrastructure to respond to emerging diseases (COVID-19) Most participants expressed interest in sustaining the program beyond the study period.	“It is possible to continue delivering the program in the new COVID-19 reality. First of all, we need HTN medications. Second, we need training to provide health coaching sessions – we already have that, and we need to strengthen it. Third, we need to continue delivering health coaching sessions, but now as part of patient clubs.” – Health area nurse, MHD3

*MHD* municipal health district

### Sustainability considerations

Table 5 includes representative quotes and a summary of meta-inferences related to program sustainability. Overall, we found that qualitative and quantitative phases across settings, participants and RE-AIM / PRISM dimensions confirmed and expanded on each other. First, we found that most participants considered that it would be possible to sustain the program beyond the study period. All the health administrators and providers recognized the program benefits and the importance of institutionalizing it within the MOH. Second, we identified threats to sustainability across health districts, such as limited implementation infrastructure or limited fit between program and health district. Future hypertension programs could enhance fit by ensuring understanding of intervention benefits prior to implementation and identifying program champions. Third, the COVID-19 pandemic exacerbated threats to sustainability, by further limiting the infrastructure, such as available human resources and medication supply chain. To continue implementing the program in the context of COVID-19, most participants indicated that it would need to undergo certain adaptations to increase fit, such as delivering health coaching sessions in a group setting, rather than individually. Finally, the ability of healthcare teams to implement program adaptations in response to COVID-19 seemed to enhance implementation delivery of the EBI and implementation strategies. Future implementation initiatives could explore if improving the ability of health district leadership to implement program adaptations could improve the program delivery and its sustainability.

## Discussion

Our mixed methods analysis of mid-course implementation and context of a new hypertension control program within Guatemala’s public primary system during the initial months of the COVID-19 pandemic led to two major conclusions. First, we documented a close interplay between the rapidly evolving context and implementation delivery + adaptations to the EBI and the implementation strategy. Second, we identified sources of unequal program implementation and potential threats to program sustainability as well as program adaptations for improving health equity and sustainability.

We confirmed the close interplay between contextual factors, EBIs, and implementation strategies pointed out by others [1]. We found that contextual factors influenced program implementation delivery + adaptations. For instance, enhancing the implementation and sustainability infrastructure by providing basic program equipment (e.g., blood pressure monitors) and hypertensive medications prior to launching the program, initially facilitated implementation delivery. In contrast, disruptions to medication supply chain during COVID-19, led to reduced implementation delivery of the EBI. In addition to influencing implementation delivery, contextual changes led to qualitative adaptations. As a response to COVID-19, healthcare providers implemented program adaptations, some of which seemed to increase the fit between context and program. For example, to overcome challenges due to transportation disruptions and social distancing, healthcare providers began delivering hypertensive medications at the village level, bringing them closer to patients. Our study showed that rapid contextual changes at the community and primary care levels led to drastic changes in implementation delivery, which spurred planned program adaptations. Such adaptations allowed the program to be delivered within the new context. Utilizing system dynamics approaches in future studies could help to understand feedback loops and dependencies between contextual factors, EBIs, and implementation strategies, as well as the points that may be leveraged to improve implementation and clinical outcomes.

Similar to other implementation assessments conducted during COVID-19, we found that the COVID-19 emergency further stretched an overburdened and under-resourced primary care system [20, 22], threatening continued program implementation and its future sustainability. However, even though resources and time allocated to the program decreased during COVID-19, certain health districts were able to bounce back to increase implementation delivery close to pre-COVID-19 levels. Understanding the factors that increased program delivery during the implementation phase may help to draw sustainability implications. For example, we identified factors that seemed to facilitate implementation delivery during COVID-19, such as MoH staff’s perception of the EBI as beneficial; essential infrastructure, such as human resources; and strong leadership and experience implementing programs for chronic diseases. Certain adaptations that helped with implementation delivery during the implementation phase should be prioritized during the sustainability phase. For instance, we learned that the implementation strategy (team-based collaborative care) may be adapted by having primary care teams (e.g., physicians and auxiliary nurses) communicate by phone multiple times a week instead of holding monthly in-person meetings. This adaptation could allow teams to change the “form” of this strategy as needed, while still meeting with its function of making team-based decisions regarding hypertension treatment. In line with a dynamic understanding of sustainability, this study suggests that assessing a program under different scenarios during its implementation phase (e.g., before and during COVID-19) may provide insights for program sustainability, such as the contextual factors and program adaptations or refinements that may be needed to sustain it.

Following calls to utilize a health equity lens in implementation assessments [7, 9], this study found that program implementation was influenced by social and structural determinants of health, such as poverty, gender discrimination, rurality, and historical discrimination against indigenous groups. While our program was designed and implemented with socially disadvantaged groups in mind (rural and Maya-indigenous populations served by the public primary care system), results suggested that program adaptations may be needed to address health disparities within subgroups. Importantly, adaptations that allow for a flexible implementation delivery may help to reach socially disadvantage groups. For example, diversifying the ways of delivering hypertensive medications (e.g., through relatives, at home, at the health post) may help to address challenges related to medication delivery. Our explicit focus on health equity in this assessment surfaced sources of health disparities and potential ways to address them through program adaptations. However, historical and broader sources of health disparities (e.g., ethnic discrimination) will not be addressed through one specific program and would require system-level or broader policy changes.

Our study has several strengths. First, we assessed program implementation over time focusing on two different phases (before and during COVID-19), which allowed us to understand how rapidly changing contextual factors led to both the EBI and the implementation strategy’s evolution. Second, we applied widely recognized implementation frameworks, RE-AIM/PRISM, coupled with an explicit focus on health equity and sustainability. Third, we utilized a mixed methods approach, interviewed participants at different levels of the health system (e.g., providers, administrators, program evaluators), and included a range of health districts, all of which allowed for a deeper understanding of the program’s implementation within the public primary care system in Guatemala. Finally, we utilized behavior-over-time graphs, a systems thinking tool, to better understand implementation delivery changes amid contextual changes. However, our results need to be interpreted considering certain limitations. First, our analysis included five of the 8 districts that were implementing the program at the time of our assessment. Our analysis aimed to describe the implementation experience in depth, rather than making generalizable inferences. For this reason, we purposefully selected health districts with the longest implementation experience that had delivered the program both before and after COVID-19 and represented a wide spectrum of implementation experiences. The other study health districts across Guatemala have many more distinct characteristics that could change their implementation experience. Additionally, this assessment did not include perspectives from program recipients (i.e., patients), although our study team has captured patient perspectives in a different analysis [23].

Conclusions Our study contributes to calls to advance our understandings of sustainability and health equity in implementation science. Our implementation assessment provides a rich empirical example of application of complementary implementation science theories and frameworks. In addition, this study provides urgently needed information on how to assess multi-component programs in settings with limited resources and under rapidly changing contexts, such as the COVID-19 pandemic. Low-resource primary care settings in Guatemala typically face changes to the context (e.g., tropical storms during hurricane season that lead to flooding or mudslides in communities). We conducted a rich mixed methods assessment of a program implementation over time, showing the close inter-relationships between context, EBI and implementation strategy, and their influences on health equity and sustainability. These findings point to the need for robust mixed methods and more rapid assessments, and systems science approaches that help understand the dynamic relationships between contextual and implementation factors over time.

### Supplementary Information

**Supplementary Material 1.**

**Supplementary Material 2.**

**Supplementary Material 3.**

## Data Availability

The datasets used and/or analyzed during the current study are available on reasonable request.

## Abbreviations

BOT graph: Behavior-over-time graph
CIIPEC: INCAP Research Center for the Prevention of Chronic Diseases
COREQ: Consolidated criteria for reporting qualitative research
cRCT: Cluster randomized control trial
EBI: Evidence-based intervention
IECS: Institute for Clinical Effectiveness and Health Policy
INCAP: Institute of Nutrition of Central America and Panama
IRB: Institutional Review Board
LMICs: Low- and middle-income countries
MIS: Modelo Incluyente de Salud
MHD: Municipal health district
MoH: Ministry of Health
PRISM: Pragmatic, Robust, Implementation and Sustainability Model
RE-AIM: Reach, Effectiveness, Adoption, Implementation Maintenance
RITA: Rapid Identification of Themes from Audio Recording
