# Supplementary material for: Assessing the implementation of a multi-component hypertension program in a Guatemalan under-resourced dynamic context: an application of the RE-AIM/PRISM extension for sustainability and health equity
Source: Implement Sci Commun. 2024 Mar 15;5:23. doi: 10.1186/s43058-024-00560-5 (PMC10941412; doi:10.1186/s43058-024-00560-5)
Supplement: Supplementary file 1 — Supplementary Material 1. [file 43058_2024_560_MOESM1_ESM.docx]

**Guía de entrevista: Directores de área de salud / distrito de salud**

| Distrito: | ID entrevista: | |
| --- | --- | --- |
| Entrevistado: | Duración entrevista: | |
| Fecha: | Notas matriz: | Grabación: |

*Desde el 2019, INCAP y el MSPAS han implementado un programa multicomponente para mejorar el control de la hipertensión en Guatemala. Su distrito NOMBRE empezó a implementar el programa en MES, 2019. Lo invitamos a participar debido a su rol como representante del programa en su DAS/DMS. El objetivo de esta entrevista es conocer cómo se ha implementado esta intervención en su distrito y otros servicios de salud para pacientes con hipertensión. Debido a que COVID-19 ha tenido un impacto en los servicios de salud, nos interesa saber cómo los servicios dentro de su DMS/DAS han cambiado debido a COVID-19 y si ha habido cambios en las actividades del programa y atención de salud para personas hipertensas.*

*Su experiencia y perspectiva es muy valiosa, ya que Ud. ha estado directamente involucrado. No hay respuestas correctas o incorrectas. Sabemos que el programa no se ha implementado de forma perfecta y queremos aprender de esto, para saber cómo podríamos mejorarlo en el futuro.*

1. Primero nos enfocaremos en la implementación del programa antes de que iniciara COVID-19. En sus propias palabras, ¿cómo describiría el programa multi-componente para el control de hipertensión?

- ¿Hasta qué punto se ha logrado integrar el programa a todas las actividades y programas del distrito? ¿En otras palabras, diría usted que el programa se volvió de las actividades que hacen en el distrito, o aún se siente como un programa separado de los demás?
- ¿Qué componentes / actividades del programa han sido más fáciles de implementar? ¿Cuáles han sido más difíciles?
  - Medicamentos: uso de algoritmo de tratamiento, abastecimiento y entrega a pacientes
  - Seguimiento a pacientes hipertensos: Medición de PA en PS y en el hogar, sesiones de consejería
  - Personal de salud: Reuniones de equipo, capacitación a personal de salud, supervisión
- ¿Ha habido personas a las que les haya costado más / facilitado más recibir el programa de hipertensión? ¿Quiénes son estas personas? ¿Qué se podría hacer para ofrecer la atención de manera más equitativa a todas las personas con presión alta del distrito? ¿Cómo se podría responder a las dificultades que unas personas tienen en recibir los servicios (consulta, medicamentos, sesiones de consejería) recomendados para hipertensión?

1. Ahora nos enfocaremos en la implementación del programa después de COVID-19. En sus propias palabras, ¿cómo describiría la implementación del programa multi-componente para el control de hipertensión en este momento, después de que inició COVID-19?
   - Medicamentos: uso de algoritmo de tratamiento, abastecimiento y entrega a pacientes
   - Seguimiento a pacientes hipertensos: Medición de PA en PS y en el hogar, sesiones de consejería
   - Otros componentes: Reuniones de equipo, capacitación a personal de salud
2. Ahora, hablemos de las diferencias antes y después de COVID-19. ¿Cuáles diría Ud. que han sido las principales diferencias en cómo se ha implementado el programa antes y después de COVID-19?
3. Tomando en cuenta la nueva realidad con COVID-19, ¿qué considera que es necesario mantener el programa a largo plazo, incluso después de que termine el estudio de INCAP?

- ¿Qué componentes/actividades podrían continuar y cuáles no?
- Dada la nueva realidad, ¿Cómo se puede ofrecer el programa a todas las personas con HTA del distrito?

1. ¿Qué es necesario para poder mantener el programa a largo plazo, después de que termine el estudio? (e.g. recursos, personal, actividades)
2. Por último, imagine que otro DMS dentro de su DAS planea implementar el programa en este momento. ¿Qué recomendaciones les daría para que logren implementarlo?

**Guía de entrevista: Evaluadores y Asistentes de investigación**

| Distrito: | ID entrevista: | |
| --- | --- | --- |
| Entrevistado: | Duración entrevista: | |
| Fecha: | Notas matriz: | Grabación: |

El objetivo de esta entrevista es aprender cómo se ha implementado la intervención multicomponente para el control de la hipertensión en el distrito [DISTRITO]. Para hacerlo más fácil, le llamaremos “la intervención”. Para iniciar, estamos entrevistando a evaluadores como usted, debido a que Uds. Han estado en contacto con participantes y personal del MSPAS y conocen bien cómo se está implementando la intervención en este distrito. Al decir “implementación de la intervención” nos referimos a qué tanto y cómo se llevan a cabo las actividades de la intervención. Sabemos que intervenciones como ésta pueden implementarse diferente a como se habían planeado al inicio y que pueden ocurrir cambios en la implementación conforme pasa el tiempo, lo cual es normal y es esperado. También sabemos que además de sus responsabilidades de recolectar datos de participantes y de servicios de salud, ud. Puede haber tenido otros roles que han sido necesarios para la implementación de la intervención. Todo esto es normal. Nos gustaría entonces conocer, desde su perspectiva, cómo han ocurrido estos cambios en la implementación de la intervención a través del tiempo y qué rol ha jugado usted en esta implementación. No hay preguntas buenas o malas, y lo que usted diga no afectará su trabajo como evaluador de INCAP.

Debido a que la pandemia de COVID-19 ha cambiado nuestro trabajo y los servicios de salud, vamos a dividir esta entrevista en dos etapas: antes y después de que se confirmara el primer caso de COVID-19 en el distrito [DISTRITO].

Para esta **primera parte**, por favor piense en el inicio de la implementación de la intervención, específicamente en el distrito [DISTRITO], en el mes de [MES].

1. ¿Cómo describiría la ***aceptación*** inicial de la intervención en este distrito?

- *Explicar si necesario:* Con “aceptación” me refiero a las opiniones, ideas, sentimientos y disposición a tomar acción para implementar o recibir la intervención.
  1. ¿Hubo coordinadores / proveedores / pacientes y familiares que aceptaran más / menos la intervención al inicio?
- *Sugerir:* Directores y administradores de área de salud y distrito de salud.
- *Sugerir*: Proveedores de salud: Médicos y enfermeros profesionales del centro de salud, auxiliares de enfermería del puesto de salud.
- *Sugerir*: Pacientes y familiares que reciben la intervención. (Énfasis en los que no son alcanzados por la intervención).
  1. En este tiempo inicial, ¿cuál fue su **rol como evaluador**?
- *Explicar si necesario:* Además de su trabajo puramente de evaluador, que incluye recolectar datos de participantes y de la intervención, algunos de ustedes han mantenido comunicación y han hecho actividades con el área, distrito o puestos de salud.

1. Ahora pensemos en las semanas que siguieron a la implementación inicial de la intervención en [DISTRITO]. ¿Cómo se **han implementado los componentes** de la intervención? Tomando en cuenta cómo se planeaba implementar la intervención, ¿qué cambios se hicieron en esta implementación?

- **Capacitación de proveedores de salud**: Recambio de personal, interés en capacitaciones, necesidad de refrescar capacitación
- **Algoritmo de tratamiento:** Disponibilidad de medicamentos anti-hipertensivos en puestos y centro de salud; Entrega de recetas y medicinas anti-hipertensivas por proveedores de salud; Uso de medicamentos por participantes.
- **Medición de PA con auditoría y retroalimentación**: Diagnóstico de HTA, seguimiento de HTA
- **Sesiones de consejería**: Frecuencia, opinión de pacientes y familiares, proveedores
- **Medición de PA en casa**: Frecuencia, quién la realiza, opinión de pacientes y familiares, proveedores
- **Equipos colaborativos de cuidado:** Frecuencia, quiénes participan, opinión de proveedores
  1. ¿Cuáles diría usted que son los componentes de intervención han sido **fáciles** de implementar?
  2. ¿Cuáles diría usted que son los componentes de intervención han sido **difíciles** de implementar?
- *Indagar:* ¿Qué ha impedido que estos se implementen? Dificultades para los pacientes / familiares, en los servicios de salud, proveedores de salud, coordinadores y administradores
  1. ¿Qué componentes les gustan más y llevan a cabo los proveedores de salud? ¿Qué componentes no les gustan y no siguen los proveedores de salud?
  2. ¿Qué componentes les gustan y siguen más los participantes y sus familiares? ¿Qué componentes no les gustan y no siguen los participantes y sus familiares?
  3. ¿Considera usted que es factible implementar los 6 componentes de la intervención se implementen como se planeó al inicio?
- *Sugerir:* Si no, ¿qué tendría que suceder para que se implemente?
- *Sugerir: ¿*Ha habido actividades que no habrían sucedido si no hubiera sido porque Ud. Hizo algo para que sucedieran?

En esta **segunda parte**, hablaremos sobre lo que ha sucedido en el [DISTRITO] desde que sucedió el primer caso de coronavirus en ese distrito, lo cual fue en el [MES].

1. ¿Qué **cambios** han sucedido en los **servicios de salud desde que la pandemia COVID-19 inició?**
   1. ¿Qué responsabilidades o actividades tienen ahora los administradores y proveedores de salud? **¿**Ha habido algunos cambios?

- *Indagar:* ¿Ha habido algún **administrador o proveedor de salud** que haya estado asignado para la intervención de hipertensión y haya **cambiado sus funciones** luego de que inició COVID-19?
  1. ¿Qué **componentes** de la intervención **siguen realizándose**? ¿Qué ha permitido que se hagan?
  2. ¿Qué **componentes** de la intervención **ya no se realizan**? ¿Qué ha impedido que se hagan?
  3. *¿*Existen características comunes en los **pacientes y familias** que continúan recibiendo más componentes de la intervención, en comparación con los que reciben menos componentes?
  4. ¿Cómo han cambiado sus **responsabilidades y actividades** de evaluador luego de que llegó COVID-19 a este distrito?

**Guía de entrevista: Nivel Central**

| Distrito: | ID entrevista: | |
| --- | --- | --- |
| Entrevistado: | Duración entrevista: | |
| Fecha: | Notas matriz: | Grabación: |

*Desde el 2019, INCAP y el MSPAS han implementado el programa multicomponente para mejorar el control de la hipertensión en Guatemala. Lo invitamos a participar debido a que ha tenido un rol importante en este programa desde el nivel central del MSPAS. El objetivo de esta entrevista es documentar cómo se ha implementado el programa desde el punto de vista del SIAS. Debido a que COVID-19 ha tenido un gran impacto en nuestras vidas, nos interesa saber si ha habido cambios en el funcionamiento de MSPAS y servicios de salud para hipertensión.*

*Para esta entrevista nos enfocaremos en las áreas de salud que iniciaron el programa primero: Baja Verapaz, Chiquimula y Zacapa.*

*Su experiencia y perspectivas son muy valiosas, ya que ha estado directamente involucrado en el programa. No hay respuestas correctas o incorrectas. Sabemos que el programa no se ha implementado de forma perfecta y queremos aprender de esto, para saber cómo podríamos mejorarlo en el futuro.*

1. Primero nos enfocaremos en la implementación del programa antes de que iniciara COVID-19. En sus propias palabras, ¿cómo describiría el programa multi-componente para el control de hipertensión?

- ¿Hasta qué punto se ha logrado integrar el programa a todas las actividades y programas del MSPAS, DAS y DMS? ¿En otras palabras, diría usted que el programa se volvió de las actividades que hacen o aún se siente como un programa separado de los demás?
- ¿Qué componentes / actividades del programa han sido más fáciles de implementar? ¿Cuáles han sido más difíciles? ¿Cuál ha sido el rol del SIAS en esta implementación?
  - Medicamentos: uso de algoritmo de tratamiento, abastecimiento de medicamentos* y entrega a pacientes
  - Seguimiento a pacientes hipertensos: Medición de PA en PS y en el hogar, sesiones de consejería
  - Personal de salud: Reuniones de equipo, capacitación a personal de salud, supervisión
- ¿Ha habido DAS o DMS a los que les haya costado más / facilitado más implementar el programa de hipertensión? ¿Cuáles son estas DAS o DMS? ¿Qué se podría hacer para ofrecer la atención de manera más equitativa en todas las DAS Y DMS?
- Según su experiencia y conocimiento del sistema de salud, considera que podría haber comunidades personas a las que les sea más difícil / facilitado más recibir el programa de HTA? ¿Quiénes son estas personas?¿Cómo se podría responder a las dificultades que unas personas tienen en recibir los servicios (consulta, medicamentos, sesiones de consejería) recomendados para hipertensión?

1. Ahora nos enfocaremos en la implementación del programa después de COVID-19. En sus propias palabras, ¿cómo describiría la implementación del programa multi-componente para el control de hipertensión en este momento, después de que inició COVID-19?
   - Medicamentos: uso de algoritmo de tratamiento, abastecimiento y entrega a pacientes
   - Seguimiento a pacientes hipertensos: Medición de PA en PS y en el hogar, sesiones de consejería
   - Otros componentes: Reuniones de equipo, capacitación a personal de salud
2. Ahora, hablemos de las diferencias antes y después de COVID-19. ¿Cuáles diría Ud. que han sido las principales diferencias en cómo se ha implementado el programa antes y después de COVID-19?

- ¿Qué factores han causado estas diferencias?

1. Tomando en cuenta la nueva realidad con COVID-19, ¿Diría usted que es posible continuar implementando el programa de HTA a largo plazo, después de que termine el estudio de INCAP?

- ¿Qué componentes/actividades podrían continuar y cuáles no?
- ¿Cuáles deberían continuar y cuáles no?
- ¿Qué necesitaría existir o suceder dentro del MSPAS para que se pueda continuar implementando aún después de que termine el estudio? (e.g. recursos, personal, actividades)

1. Dada la nueva realidad con COVID-19, ¿Sería posible expandir el programa a otras áreas de salud? ¿Sería posible diseminar a todo el país?

- ¿Qué se necesitaría para lograr esto?
- ¿Cuáles serían algunas barreras para lograrlo? ¿Cuáles serían algunos facilitadores para lograrlo?
- ¿Qué implicaciones habría para el sistema de salud? (recursos humanos, servicios médicos, sistemas de información, finanzas, medicamentos, liderazgo)
