# Supplementary material for: Assessing the implementation of a multi-component hypertension program in a Guatemalan under-resourced dynamic context: an application of the RE-AIM/PRISM extension for sustainability and health equity
Source: Implement Sci Commun. 2024 Mar 15;5:23. doi: 10.1186/s43058-024-00560-5 (PMC10941412; doi:10.1186/s43058-024-00560-5)
Supplement: Supplementary file 2 — Supplementary Material 2. [file 43058_2024_560_MOESM2_ESM.docx]

| **Matrix topic** | **Definition** |
| --- | --- |
| Initial program implementation | Description of adoption of core program component and implementation strategies during the first month of the program |
| Program implementation  Before COVID-19 | Description of the implementation of core program component and implementation strategies before COVID-19 |
| Dynamic context:  Community changes during COVID-19  (PRISM) | Community factors that underwent changes during COVID-19, including transportation, lockdowns, community organization, community reactions to COVID-19. |
| Dynamic context:  Organizational changes during COVID-19  (PRISM) | Organizational factors that underwent changes during COVID-19, including reallocation of staff, disruptions to supply chain, health service suspensions, additional COVID-19 responsibilities, etc. |
| Program implementation  during COVID-19 | Description of implementation (delivery + adaptations) of core program component and implementation strategies during COVID-19, similarities and differences compared to pre-COVID-19 |
| Implications for health equity | Difficulties reaching or implementing program at the patient- and healthcare-facility level from the perspective of participants; subgroups of participants less reached; healthcare facilities with difficulties to implement program; contextual changes leading to larger difficulties to reach / implement program; program adaptations that may facilitate reaching subgroups of participants. |
| Implications for sustainability | Intention to sustain program beyond study period and recommendations for doing so; contextual changes that threatened program implementation and may threaten program sustainability; program adaptations that may facilitate program sustainability over time. |
| MHD- Municipal health district; HP – Health post; HC – health center; HTN – Hypertension; HTZ – HTN medication  Participants: RA – INCAP Research assistant; EV – INCAP field data collector; | |
